# Patient- and Provider-Reported Experiences of a Mobile Novel Digital Therapeutic in People With Opioid Use Disorder (reSET-O): Feasibility and Acceptability Study

**DOI:** 10.2196/33073

**Published:** 2022-03-25

**Authors:** Sarah Kawasaki, Sara Mills-Huffnagle, Nicole Aydinoglo, Halley Maxin, Edward Nunes

**Affiliations:** 1 Department of Psychiatry and Behavioral Health Penn State Health Hershey, PA United States; 2 Department of Psychiatry Columbia University New York, NY United States

**Keywords:** reSET-O, digital therapeutic, opioid use disorder, prescription digital therapeutic

## Abstract

**Background:**

Medications for the treatment of opioid use disorder, such as buprenorphine, are effective and essential for addressing the opioid epidemic. However, high dropout rates from medication remain a challenge. Behavioral treatment with contingency management and cognitive behavioral counseling has shown promise for improving the outcomes of buprenorphine treatment but is complicated to deliver. The delivery of behavioral treatment through technology-based platforms has the potential to make it more feasible for widespread dissemination.

**Objective:**

reSET-O is a prescription digital therapeutic and a commercial adaptation of the Therapeutic Education System, an internet-based program with a Community Reinforcement Approach to cognitive behavioral therapy. It delivers cognitive behavioral therapy modules and contingency management rewards upon completion of modules and negative urine drug screens. This pilot study aims to assess the feasibility and acceptability of reSET-O in a community-based opioid treatment program with a Hub and Spoke model of care as part of a larger strategy to maintain individuals in treatment. Objective and qualitative results, as well as acceptability and likeability of reSET-O, were obtained from 15 individuals.

**Methods:**

English-speaking individuals aged ≥18 years with a diagnosis of current opioid use disorder were recruited after being on buprenorphine for at least 1 week of treatment. Two 12-week prescriptions for reSET-O were written for the 24-week study. Patient reports of drug use and likeability scales of reSET-O were conducted at weeks 4, 8, 12, and 24 of the study. Qualitative interviews were also conducted. A total of 4 providers were recruited and provided feedback on the acceptability and feasibility of reSET-O.

**Results:**

Of the 15 participants who participated in this pilot study, 7 (47%) completed 24 weeks, and 8 (53%) were unable to complete because of dropout after enrollment, attrition in treatment, or incarceration. An average of US $96 in contingency management rewards were earned by participants for the completion of modules for the duration of the pilot study. Participants’ subjective feedback revealed that reSET-O was easy to use, enjoyable, and helped provide a safe space to admit recurring substance use.

**Conclusions:**

reSET-O was well accepted based on patient and provider feedback in this pilot study; however, adherence and retention in treatment remain areas for improvement. Randomized control trials are needed to assess whether retention of community-based buprenorphine treatment is enhanced through the use of technology-based behavioral interventions such as reSET-O.

## Introduction

The opioid epidemic has spread rapidly over the past decade, reaching virtually every region of the United States. Approximately 2.4 million Americans are currently addicted to opioids, and the prognosis is poor; if left untreated, the risk of death from opioid overdose is high [1]. Although different types of medications for opioid use disorder (MOUDs), such as buprenorphine–naloxone, buprenorphine, methadone, and extended-release naltrexone, may be remarkably effective if patients adhere to treatment, adherence to treatment remains challenging [2]. Retention rates in treatment range from 10% to 60%, depending on the medication type [3-14]. In addition to the challenges in adherence, challenges in accessing MOUD present an obstacle not easily surmounted. Methadone can only be dispensed under special regulations that are burdensome to patients (eg, daily attendance at a clinic is initially required). Buprenorphine, available by prescription from any licensed physician who, up until recently, has completed an 8-hour waiver training, has struggled to penetrate primary medical care settings. The supply of physicians, nurse practitioners, and physician assistants prescribing buprenorphine remains limited, and the use of a buprenorphine waiver to the maximum extent of allowed patients is underused [15]. A combination of universally available MOUD along with strategies to combat MOUD treatment attrition and adherence is critical to combat the opioid epidemic.

A key systemic barrier that has been identified is the lack of access to behavioral intervention and counseling to accompany MOUD prescribing [16-18]. Behavioral interventions have the potential to address poor adherence to medication. The provision of counseling is a regulatory requirement for methadone, buprenorphine, and buprenorphine–naloxone treatment. Furthermore, evidence suggests that counseling and behavioral treatments improve the adherence to and effectiveness of MOUD [19], particularly with contingency management (CM) approaches [20-22]. Primary care practices and other clinical settings that are new to addiction treatment typically lack staff with expertise in relevant behavioral treatments, and this gap in care contributes to a reluctance to treat patients with opioid use disorder (OUD) in this setting [16]. Even specialty addiction treatment programs may struggle to deliver more than rudimentary counseling because of time constraints and a lack of expertise in the latest evidence-based interventions. Different models of care have been implemented to address this with success. Vermont established a Hub and Spoke system [23] meant to provide support for practices that may have had barriers to successful outcomes. In this system, the *hub* acts as the specialty treatment center, initiating or escalating OUD medication treatment quickly with the ability to provide the most intense care, including MOUD and therapy, whereas *spoke* sites are primary care practices that may continue patient MOUD after stabilization. Penn State Health partnered with an opioid treatment program (OTP) at the Pennsylvania Psychiatric Institute to use State-Targeted Response funds to establish a Hub and Spoke program [24]. This has allowed for the coordinated care and expansion of MOUDs based in Harrisburg, Pennsylvania, and surrounding counties, including rural regions in south-central Pennsylvania.

Despite the implementation of Hub and Spoke systems, challenges related to behavioral interventions persist, including limited counselor capacities at *hub* sites and potentially no counseling services at *spoke* sites. Mobile apps that deliver behavioral interventions may be beneficial to OUD treatment, helping to fill the gap in the provision of behavioral interventions [25,26]. reSET-O, generated by Pear Therapeutics, Inc, is a comprehensive cognitive behavioral treatment delivered through a mobile phone–based app, with an evidence base suggesting that it can improve the adherence and outcome of MOUD treatment for OUD compared with standard counseling alone [27]. reSET-O is derived from the Therapeutic Education System (TES), developed by academic investigators as a web-based tool delivered by interacting with a computer, delivering a combination of CM—modest monetary or nonmonetary rewards for completion of therapy modules and producing negative drug urine—with cognitive behavioral counseling based on the Community Reinforcement Approach (CRA). reSET-O is a commercial version of TES, adapted for marketing and widespread use, and is delivered as a mobile-based app. reSET-O is available through prescription, and the cost is intended to be covered by insurance. Importantly, the cost of contingent rewards is built into the third-party reimbursement.

CM has been well-established as effective for the treatment of substance use disorders (SUDs) [20,28]; however, funding for incentive rewards has been almost exclusively provided by research grants. The problem of how to fund contingent rewards has stymied the application of CMs in real-world treatment. Thus, the fact that reSET-O can fund incentives by bundling costs into third-party payments is an important advancement. Cognitive behavioral counseling delivered by reSET-O is modeled after the CRA, focusing on cognitive behavioral strategies to achieve abstinence from drugs and build a healthy lifestyle. As approved by the Penn State Health College of Medicine institutional review board, this study piloted the feasibility and acceptability of reSET-O in conjunction with buprenorphine management to (1) assess how individuals in treatment in this Hub and Spoke clinic would interact with this novel intervention and (2) inform future, larger controlled trials using reSET-O.

## Methods

### Overview

An uncontrolled, 24-week pilot feasibility and acceptability study that added reSET-O to standard care for patients with OUD initiating buprenorphine in an OTP and serving as the hub in a Hub and Spoke system was conducted. The *hub* provides both methadone and buprenorphine treatment in an outpatient clinic specializing in medication treatment for OUDs. reSET-O was prescribed for a 12-week period and was then renewed for a second 12 weeks for each participant to reach a total prescription duration of 24 weeks. English-speaking individuals aged ≥18 years who were on buprenorphine treatment for at least 1 week were eligible to participate and enroll in the pilot study.

**Figure 1:**
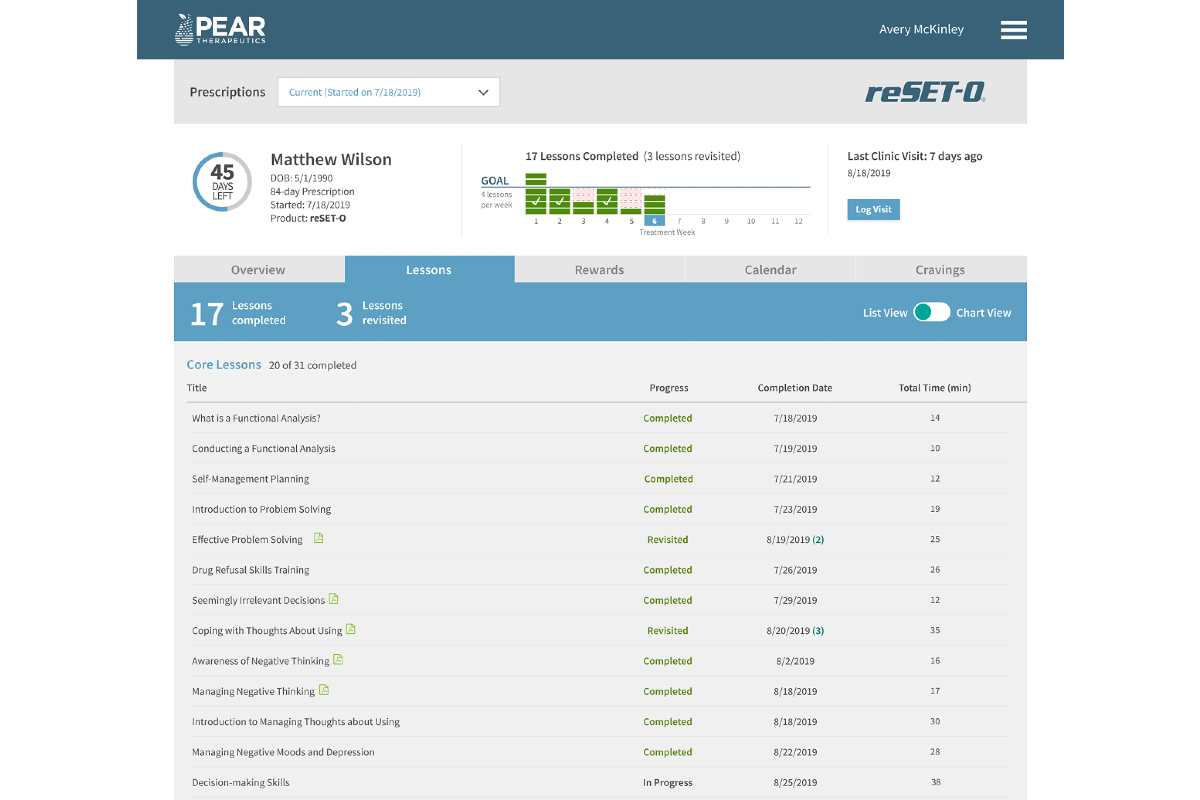
reSET-O clinician dashboard and participant lesson progress. This screenshot is an example of the clinician dashboard while viewing an enrolled participant’s progress with reSET-O modules. Note that only some of the reSET-O modules are shown in this screen shot. Provided is the title of the module; whether the module was completed, revisited (eg, completed more than once), in progress, or not completed (not pictured); date of completion; and the total amount of time spent on the module. For modules not completed, the Completion Date and Total Time (min) columns would be blank. Copyright 2022 Pear Therapeutics (US), Inc. All rights reserved. Used with permission. Not real patient data.

**Figure 2:**
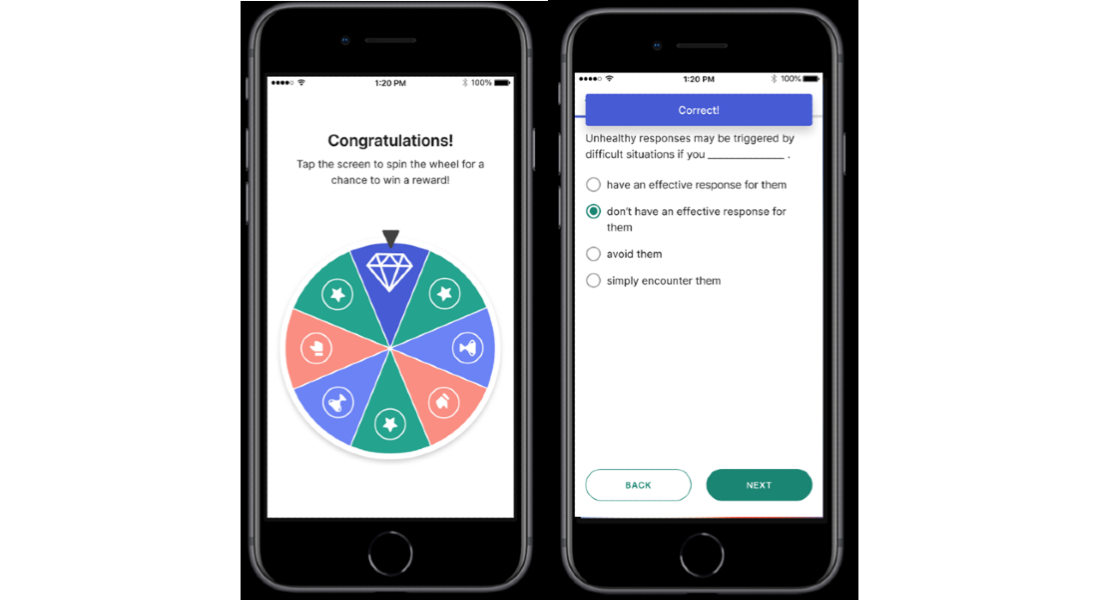
reSET-O participant quiz question and reward screens. This is an example of the type of quiz question that a participant could see (right) while using reSET-O, as well as when the participant was eligible and prompted to spin the wheel (left) to earn a reward of either a monetary amount applied to a specified gift card vendor or written reinforcement. Copyright 2022 Pear Therapeutics (US), Inc. All rights reserved. Used with permission. Not real patient data.

**Figure 3:**
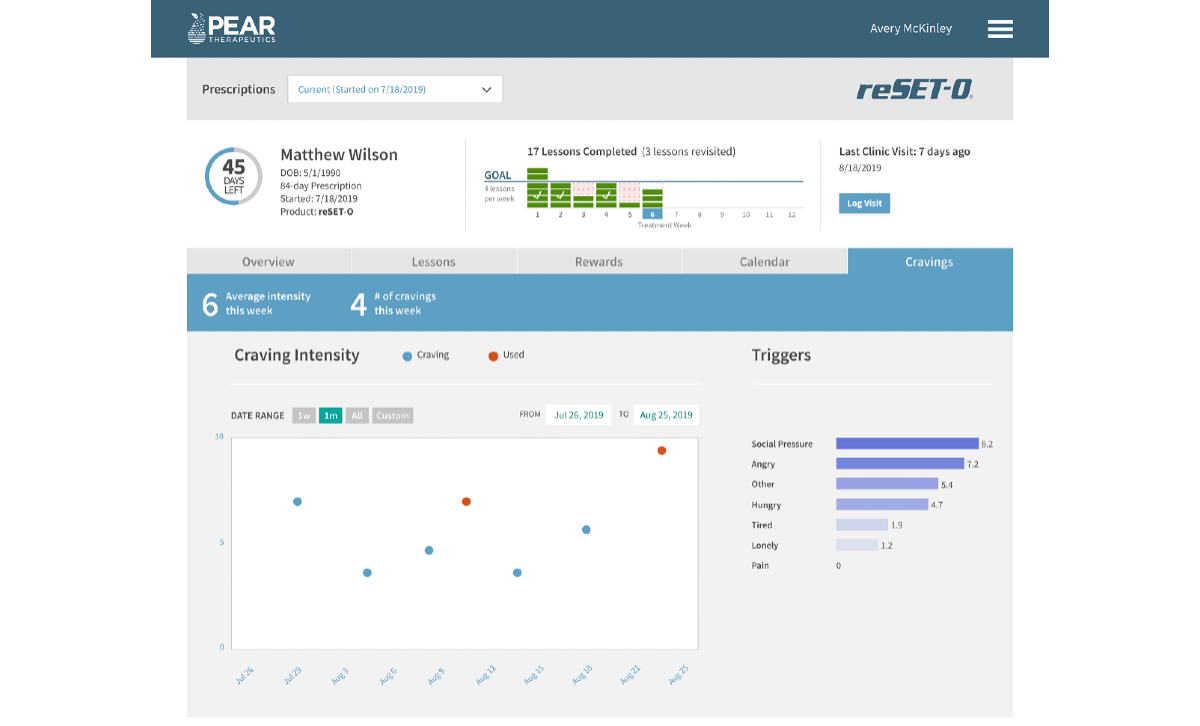
reSET-O clinician dashboard and participant reported cravings. This screenshot is an example of the reSET-O clinician dashboard when viewing a participant’s reported cravings while enrolled with reSET-O. After a participant opens reSET-O, they are asked to report any cravings and use, as well as follow-up questions related to craving intensity and potential triggers to use drugs. Copyright 2022 Pear Therapeutics (US), Inc. All rights reserved. Used with permission. Not real patient data.

**Figure 4:**
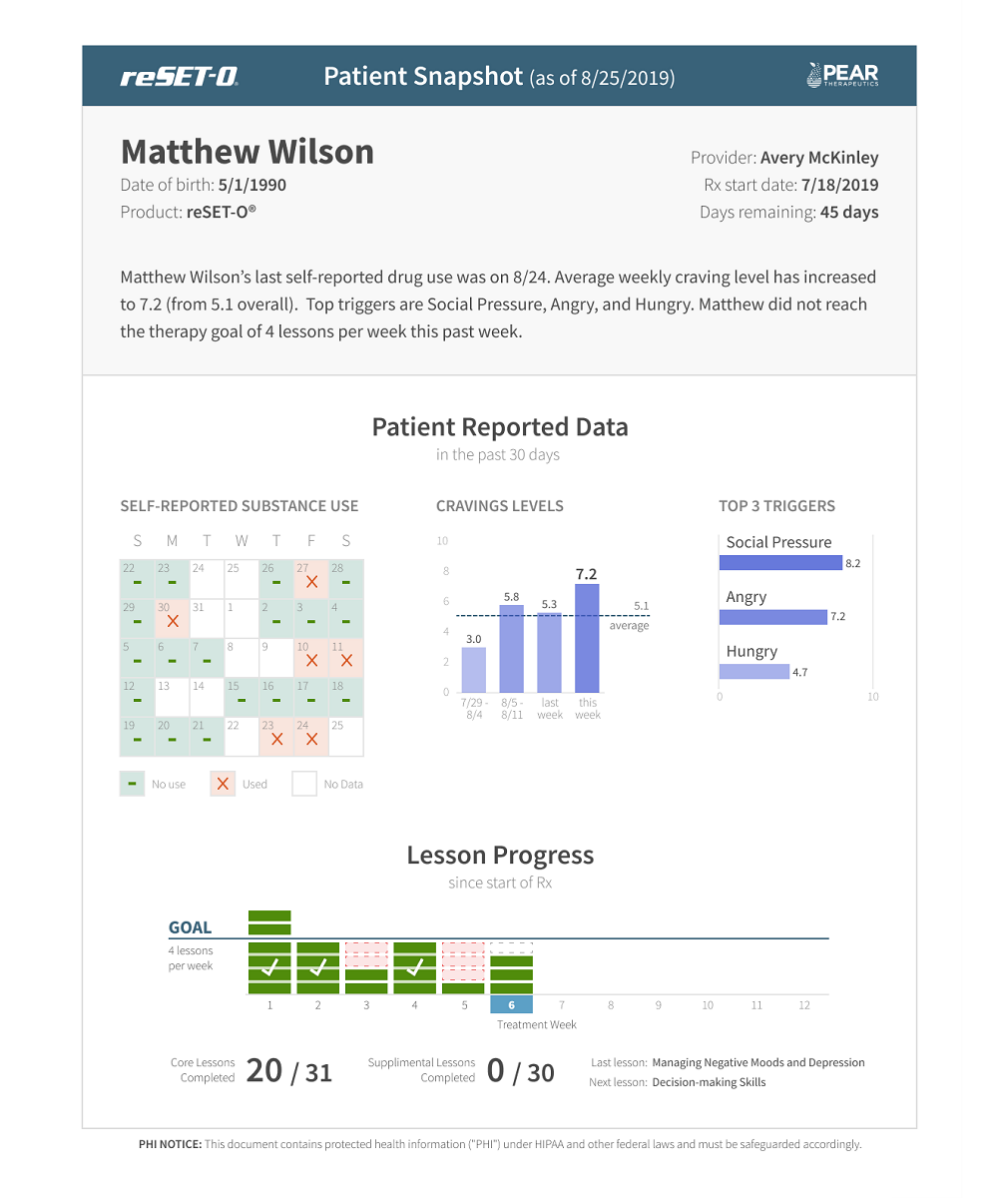
reSET-O clinician dashboard summary and patient snapshot. This is a screenshot of the clinician dashboard while viewing a participant’s summary or snapshot data. This information includes days when drug use was self-reported, brief information related to cravings, and the number of lessons completed, stratified by week. Copyright 2022 Pear Therapeutics (US), Inc. All rights reserved. Used with permission. Not real patient data.

reSET-O is a commercially available prescription digital therapeutic that delivers interactive, self-paced psychoeducational therapeutic modules regarding substance use and is based on the TES. TES is a web-based program that provides written, auditory, and video modules that instill cognitive behavioral skills based on a CRA. The CRA is a therapeutic system that teaches coping skills for staying off drugs and builds skills and activities that are consistent with a healthy lifestyle. The topics of these modules range from managing triggers of substance use and building healthier coping and social skills to HIV prevention and reducing high-risk sexual behavior (see Figure 1 for examples of topics as displayed on the clinician dashboard). Modest monetary rewards (such as gift cards) or nonmonetary rewards are earned for module completion and when negative urine drug screens (USDs) are achieved (see Figure 2 for an example of the reward programming viewed by the participant). With the commercial release of reSET-O, new additions included contingent rewards that may be delivered in the form of gift cards to retail outlets, with the cost covered by third-party insurances. Furthermore, reSET-O prompts daily check-ins, beginning with in-app reports of substance use and cravings, as indicated by the participant (see Figure 3 for an example of endorsed cravings as displayed on the clinician dashboard). Finally, the Patient Services Center, currently referred to as PearConnect, created by Pear Therapeutics, the makers of reSET-O, is a call center that connects patients to a dedicated advocate for support throughout their treatment and prescribers and health care providers to clinician dashboard resources. If a lower activity of reSET-O is detected, Patient Services Center representatives can communicate with patients on a weekly basis to troubleshoot any technical difficulties. reSET-O includes a web-based provider dashboard that permits treatment providers to examine participation and patient engagement remotely (see Figure 4 for an example of a participant snapshot as displayed on the clinician dashboard), as well as facilitate future patient-provider clinic discussions on lessons learned from the modules completed.

### Ethics Approval

Institutional review board approval was obtained for this pilot study from Penn State College of Medicine, under study number 9931 on November 27, 2018, and procedures were followed in accordance with the ethical standards of the institutional committee on human experimentation and with the Helsinki Declaration of 1975, as revised in 2000.

### Patient Participants

Eligible individuals were aged ≥18 years; able to read, write, and comprehend English; had a diagnosis of OUD, as determined through routine clinical evaluation; initiated treatment at a community-based OTP serving as the *hub* in a Hub and Spoke system of care; and were prescribed buprenorphine for OUD. We excluded any individuals engaging in outpatient detoxification or needing a higher level of care, such as inpatient or residential treatment, as well as those not desiring MOUD. Individuals were invited to join the pilot study during their medical appointments and consented in the clinic if interested. All participants provided written informed consent.

### Procedures

After obtaining participant consent, reSET-O was prescribed to participants for 12 weeks at a time, with a total of 2 prescriptions (24 weeks of consecutive treatment). reSET-O asks participants to log onto the app and complete 4 learning modules per week, each focusing on a particular skill. The modules are presented in a fixed order, beginning with modules on skills to avoid drugs, followed by modules on building a healthy lifestyle and HIV risk reduction. Each completed module yields a chance to earn rewards in the form of gift cards to retail outlets. Participants were evaluated via the self-report of drug use with the Timeline Follow-Back (TLFB) method, USDs, and three mood assessments: the Kessler-10 [29,30], the Posttraumatic Stress Disorder (PTSD) Checklist–Civilian [31-33], and the Patient Health Questionnaire-9 [34]. These evaluations occurred at baseline and at 4, 8, 12, and 24 weeks after study entry. Self-reported drug use was collected using TLFB [35,36] and verified using USDs. Participants were scored as not abstinent at a visit if either a self-report or a USD was positive for opioids. Within the reSET-O, craving assessments were performed throughout the duration of the prescription.

Participant feedback data regarding reSET-O was collected on the Intervention Acceptability Feedback Form (IAFF), evaluating seven characteristics—interest, usefulness, new information learned, ease of understanding, relevancy, satisfaction, and likeability—on a Likert scale of 0 to 9 (0=lowest and 9=highest). The IAFF was completed at each visit after baseline (weeks 4, 8, 12, and 24).

Qualitative interviews with participants were conducted by research assistants between weeks 8 and 12 to capture information from participants about their experience using the app, acceptability of the app, and suggestions for improvement. Approximately 60% (9/15) of the participants completed the interviews. Each participant was asked 9 standard questions (Textbox 1); then, the staff member could ask follow-up and probing questions as needed to elicit additional information. The interviews lasted between 30 and 45 minutes, were digitally audio recorded, and transcribed verbatim for analysis purposes.

A total of 3 research staff members reviewed all 9 interviews, extracting transcribed text relevant and responsive to the domains of satisfaction or dissatisfaction and the likeability of the intervention. Responses to the following research question: “How did participants like using reSET-O?” were categorized into unique themes, which were reviewed and discussed by a larger research team to reach a consensus.

**Qualitative interview questions and examples of follow-up questions.**

**Tell me how you use technology in your life.**

- What other apps do you use regularly?
- How often do you use other apps?
- Where do you get information about health and/or medical questions?

**Think about the times you used the reSET-O app.**

- At what times during the day did you use the app? How often?
- When did you access the reSET-O app the most?
- Where did you access the reSET-O app?
- How often did you check in with the app between modules?

**What interested you about this study?**

- Why did you decide to participate in the study?
- How did it fit in with your counseling at the clinic?
- Did you talk about the reSET-O app with other people (friends, family, or other clinic patients)?

**How did you like using the reSET-O app?**

- Can you give me an example of what you liked about it?
- What did you not like?
- Would you recommend this app to someone? Why or why not?

**How useful/relevant to your life was the reSET-O app?**

- Which modules were most useful and/or relevant?
- Which were the least useful and/or relevant? Why did those not work for you?
- Did you repeat any of the modules? Which ones? Why?
- Which features of the app were most useful and/or relevant?
- Which features were the least useful and/or relevant? Why did those not work for you?

**Is there anything about the reSET-O app that you would change?**

- Content?
- Language?
- Videos?
- Examples?
- What type of changes would you make?

**What else would you like the reSET-O app to do?**

- Give examples of additional features or module topics not currently available.

**Were you able to complete all the study tasks up to this point?**

- Was there anyone or anything that helped you complete all of the research study tasks up to this point?
- If not, why were you not able to complete all of the study tasks up to this point?
- Was there anyone or anything that helped you complete some of the research study tasks up to this point?
- Was there anything about the research study that kept you from completing all the study tasks?
- Was there anything about using the reSET-O app that made you not want to continue in the study?
- What was the main reason for you to continue in the study?
- Was there anything else about the research study that made you not want to continue?

**Were you able to complete all or some of the reSET-O learning modules up to this point?**

- Was there anyone or anything that helped you complete all of the learning modules up to this point?
- If not, why were you able to complete all of the learning modules up to this point?
- Was there anyone or anything that helped you complete some of the learning modules up to this point?
- Was there anything about the reSET-O app that kept you from completing all of the learning modules?
- Was there anything about using the reSET-O app that made you not want to continue the learning modules?
- What was the main reason for you to continue using the app (if applicable)?
- Was there anything else about the research study or the app that kept you from completing the reSET-O modules?

### Provider Participants

Provider data regarding the acceptability and feasibility of reSET-O were also collected. Clinicians working in the treatment programs were asked to create their own reSET-O accounts and review the learning modules. After 3 weeks of reSET-O use, data from 4 clinical providers were collected through the Weiner Intervention Acceptability, Appropriateness, and Feasibility form (WIAAF; Multimedia Appendix 1), evaluating the acceptability, appropriateness, and feasibility of the app intervention. Each category was assessed through 4 prompts, each using a 5-point Likert scale (1=lowest and 5=highest). The highest score for any given category could be 20. The WIAAF, currently in development, has no cutoff scores for interpretation; however, higher scores indicate greater acceptability, appropriateness, or feasibility.

## Results

### Overview

Of the 15 participants, 3 (20%) female patient participants and 12 (80%) male patient participants with OUD were enrolled in the study, with an average age of 36.2 (SD 9.3) years (Table 1). Approximately 73% (11/15) of the participants identified as White, whereas 7% (1/15), 13% (2/15), and 7% (1/15) identified as Black or African American, biracial, or other, respectively, and 13% (2/15) identified as Hispanic (Table 1). Of these 15 participants, 2 (13%) withdrew consent after baseline; 3 (20%) participants dropped out of clinical care before week 8 and were unable to be reached, and another 3 (20%) became incarcerated between weeks 12 and 24 during the study. Thus, of the 15 participants, 8 (53%) withdrew and/or were unable to complete the study, and 7 (47%) were able to complete all 24 weeks of the study.

**Table 1:** Study demographics (N=15).

Characteristics			Baseline (n=15)		Week 4 (n=11)		Week 8^a^ (n=10)		Week 24 (n=7)
Age (years), mean (SD)			36.2 (9.3)		37.4 (9.4)		37 (9.1)		41.4 (8.1)
**Gender, n (%)**									
	Male	12 (80)		11 (100)		10 (100)		7 (100)	
	Female	3 (20)		0 (0)		0 (0)		0 (0)	
**Race, n (%)**									
	White	11 (73)		8 (73)		7 (70)		5 (71)	
	Black or African American	1 (7)		1 (9)		1 (10)		1 (14)	
	Biracial	2 (13)		2 (18)		2 (20)		1 (14)	
	Other^b^	1 (7)		0 (0)		0 (0)		0 (0)	
**Ethnicity, n (%)**									
	Hispanic	2 (13)		1 (9)		1 (10)		14	

^a^At weeks 8 and 12, the same 10 participants remained in the study.

^b^Participants identified as Hispanic only.

### Patient Participant Feedback and Outcomes

#### Clinical Outcomes

Although all 15 participants had current opioid use at the time of entry into the clinic, at the point of entry into the study, 8 (53%) had already achieved abstinence from opioids through outside treatment, whereas 7 (47%) participants reported and tested positive for opioid use. Of the 7 participants who reported and/or tested positive for opioid use at baseline, 1 (14%) endorsed opiate use at weeks 4, 8, and 24, and 3 (43%) endorsed opioid use at week 12 (Table 2). Of the 8 participants who did not report or test positive for opioid use at baseline, none endorsed opioid use at weeks 4, 8, and 24, whereas 1 (13%) endorsed opioid use at week 12 (Table 2). Of the 15 participants, 7 (47%) participants completed the TLFB at all time points; however, 1 (7%) participant had trouble urinating at some appointments because of other medical conditions. In this case, only TLFB was used to assess abstinence.

Current symptoms of emotional distress were measured using the self-report screening instruments for depression (Patient Health Questionnaire-9), general emotional distress (Kessler-10), and PTSD symptoms (PTSD Checklist–Civilian were collected at all 5 time points. Means and SDs were calculated and are reported in Table 3. Compared with the baseline, the mean scores decreased over time across all measures, although the scores reflected, at most, mild levels of severity from baseline through the follow-ups.

In application, patient-reported and -endorsed cravings and craving triggers were collected throughout the duration of the reSET-O prescriptions and reported as frequency counts (Table 4). Approximately 53% (8/15) of patients reported cravings within reSET-O. Overall, by the end of the trial, there were fewer reports of endorsed cravings and craving triggers when compared with the beginning of the trial; however, 13% (2/15) of participants continued to report cravings and subsequent triggers throughout both of the reSET-O prescriptions.

**Table 2:** Opioid positive and negative results based on Timeline Follow-Back and urine drug screens (N=15)^a^.

Opioid status at baseline	Baseline (n=15)	Week 4 (n=11)	Week 8 (n=10)	Week 12 (n=10)	Week 24 (n=7)
Participants testing *positive* at baseline (positive for opioids), n/N (%)	7/7 (100)	1/4 (25)	1/4 (25)	3/4 (75)	1/1 (100)
Participants testing *negative* at baseline (positive for opioids), n/N (%)	0/8 (0)	0/7 (0)	0/6 (0)	1/6 (17)	0/6 (0)

^a^Patients were scored as negative if both self-report for the past 30 days by Timeline Follow-Back was negative for opioids and urine toxicology was negative for opioids and scored as positive otherwise.

**Table 3:** Negative mood scores.

Time point	Participants, n (%)	PHQ-9^a,b^, mean (SD)	Kessler-10^c^, mean (SD)	PCL-C^d,e^, mean (SD)
Baseline	15 (100)	10.33 (6.93)	21.66 (10.76)	17 (14.73)
Week 4	11 (73)	6.46 (6.19)	15.86 (11.03)	14.73 (13.23)
Week 8	10 (67)	5.06 (5.49)	14.66 (11.27)	11.86 (10.84)
Week 12	10 (67)	5.93 (5.79)	14 (11.55)	12.6 (11.87)
Week 24	7 (47)	4.53 (5.57)	10.86 (12.22)	11.6 (14.20)

^a^PHQ-9: The Patient Health Questionnaire-9.

^b^Scores range from 0 (minimal) to –27 (severe) depression asking, “over the last 2 weeks, how often have you been bothered by any of the following problems?” Scores in the 5 to 9 range are considered mild severity.

^c^The Kessler-10 Psychological Distress Scale asks participants how they have been feeling over the past 1 month. Scores range from 10 (minimal) to 50 (severe distress).

^d^PCL-C: Posttraumatic Stress Disorder Checklist–Civilian.

^e^The PCL-C asks, “how much have you been bothered by each of the following 20 statements in the past 1 month?” Scores range from 0 to 80, with scores >30 being likely to have a diagnosis of posttraumatic stress disorder.

**Table 4:** In-app patient-reported triggers to endorsed cravings (n=8 respondents)^a^.

Category	Pain	Anger	Other	Tired	Hungry	Lonely	Social pressure
Patients reporting triggers (first 12 weeks), n (%)	3 (38)	3 (38)	5 (63)	5 (63)	2 (25)	6 (75)	0 (0)
Patients reporting triggers (second 12 weeks), n (%)	1 (13)	0 (0)	1 (13)	2 (25)	0 (0)	1 (13)	0 (0)

^a^Each reSET-O check-in featured craving assessment and triggers that induced cravings to use drugs: pain, anger, fatigue, hunger, isolation, and social pressure.

#### reSET-O Patient Feedback

After the first 12 weeks, 73% (11/15) of participants had accessed reSET-O, with an average lesson completion of 15.7 lessons, ranging from 0 to 36 completed lessons. The rewards earned during this time averaged US $50, ranging from US $5 to US $120. Approximately 33% (5/15) of participants engaged with reSET-O throughout the 24 weeks of the trial. Among these participants, there was an average lesson completion of 15.2 lessons, ranging from 0 to 37 completed lessons, and an average of US $49 rewards earned, ranging from US $0 to US $85.

It should be noted that individuals who completed all 24 weeks of the study completed, on average, 6.2 lessons per week and repeated modules. Those who dropped out before 12 weeks completed an average of 2.5 lessons per week.

**Figure 5:**
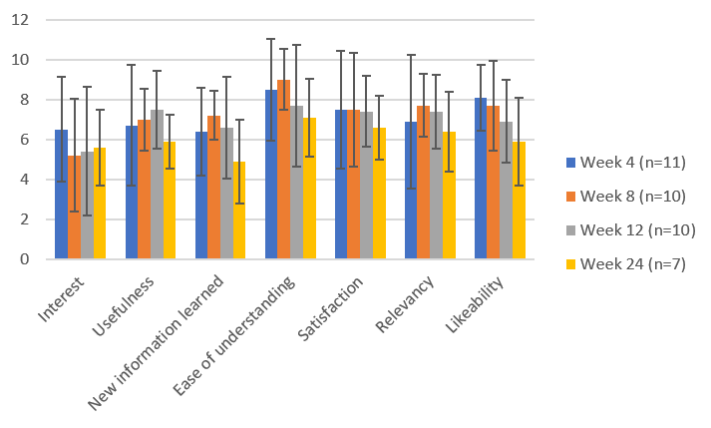
Average participant Intervention Acceptability Feedback Form scores. Participants were asked to rate the reSET-O in each of the 7 categories listed on the x-axis. Overall, the scores fluctuated in the range of 5 to 9 out of a maximum possible score of 9, suggesting moderately good acceptability on all dimensions. Scores appeared to diminish over time, especially during week 24.

Likeability scores, as assessed by the IAFF, are shown in Figure 5. The IAFF comprises 7 categories, including likeability, which was assessed using a Likert scale ranging from 0 to 9. Anchors were generated based on the prompt. For example, for the likeability category, a score of 0 indicated *I do not like it at all*, and a score of 9 indicated *I like it a lot*. With regard to likeability, after 4 weeks of reSET-O use, the average scores ranged between 6 and 8 out of a maximum possible score of 9 across all categories. The overall trend of scores appeared to drop slightly by the end of the 24 weeks, with an average range of 4.9 to 7.1 across all categories (Figure 5).

Qualitative feedback indicated overall reSET-O feasibility and acceptability by participants, as well as suggestions for improvement. The first identified theme was motivation and attitude regarding treatment delivery (eg, delivery similar to a provider appointment). Participant 3 illuminated this theme stating the following:

I also like that the app [reSET-O®] is more personal, I feel like I can be open and honest because it’s a person but not a person. I feel like I can say things or endorse things in the app [reSET-O®] that I normally wouldn’t say or want to talk about in a group setting

participant 3, male, 45 years

The second identified theme was relevancy (eg, content relevant to personal use or life). Participant 9 expressed the following:

time management and anxiety. I think there was one on depression and anxiety stuff like that, relates to me

participant 9, male, 30 years

The third theme was app features (eg, ease of use). Participant 11 stated the following:

the general app [reSET-O®], you know, the bones of it are good, you know. It takes you where you need to be, and it’s very easy to use.

participant 11, male, 40 years

The last theme was the impact on knowledge, skills, and behavior (eg, module content). Participant 8 expressed the following:

People that actually want to stay clean, it [reSET-O®] actually gives you different information that you never knew.

participant 8, male, 30 years

Many participants reported liking the rewards earned, the novelty of the treatment, and its similarity to provider treatments. Some participants found the content relevant to their own situations, and most felt that reSET-O was very easy to use. Additional analyses demonstrated that, overall, reSET-O felt personal to participants with relevant content and ease of use.

Areas of improvement were also suggested by participants during the qualitative interviews. Reported problematic areas with reSET-O included varying individual relatability to any module, as noted by a participant that the modules sometimes “felt like a drag,” and a burdensomely persistent notification system if a participant did not use the app by an expected time point.

#### reSET-O Provider Feedback

Provider feedback was collected on the WIAAF after 3 weeks of reSET-O use to gauge provider insights on the acceptability, appropriateness, and feasibility of reSET-O; the average score for the acceptability of reSET-O was 13.8 (SD 2.1), whereas appropriateness and feasibility were 12.5 (SD 2.5) and 15.8 (SD 0.5), respectively. Each category was assessed by 4 prompts, which were measured using a Likert scale ranging from 1 (completely disagree) to 5 (completely agree), and these scores were summed for a maximum score of 20. Although the WIAAF does not have established or validated cutoff scores for interpretation, the higher the score, the greater the acceptability, appropriateness, and feasibility. These scores suggest acceptability and feasibility in the good range but are short of the maximum.

Qualitative feedback from providers suggested that providers liked reSET-O and saw its potential as being implemented in a clinical setting. However, areas for improvement were also noted. Some providers indicated that the content and examples felt as though they were written by someone who had not experienced substance use and recovery. For example, providers expressed that some of the language used was not at an appropriate reading level and that the language used was stigmatizing. For instance, the phrase *beating drug addiction* was reported as pejorative, as if substance use was simply something to overcome and move on with one’s life as opposed to a chronic illness, for which “life changes and long-term commitment [were] needed to sustain recovery,” as quoted by an individual. Providers also indicated that it would be useful to have an alert option within reSET-O to alert a provider or recovery support person when a patient is in an emergency situation, in distress, or in need of support. It was also suggested that when starting a new module, a review of the last completed module should occur before beginning the new module content.

## Discussion

### Principal Findings

In this preliminary feasibility pilot study among patients undergoing buprenorphine treatment for OUD, reSET-O appeared generally acceptable and feasible, warranting further study. However, there was still a high rate of dropout, and the extent to which patients used the app varied. Some patients used the learning modules and appreciated the cognitive behavioral skills covered, whereas others did not engage. Opioid use was low among patients who remained in the study, although many of these patients had already achieved abstinence at the study baseline. Depression and anxiety symptoms were within the mild range of severity, with trends toward improvement. In addition, across all 24 weeks, the participant IAFF satisfaction ratings were between 5 and 9, out of a total of 9, for the seven assessed areas (interest, usefulness, new information learned, ease of understanding, satisfaction, relevance, and likeability), suggesting a good level of satisfaction. These scores decreased slightly over time for all assessed categories, which may be a reflection of the decay in enthusiasm over time. There was a high rate of dropout from the study, with 91% (10/11) of the patients who initiated reSET-O completing 12 weeks of reSET-O and 64% (7/11) completing 24 weeks. This is perhaps not surprising, given the general problem of attrition from apps, as well as the high rates of dropout from the treatment characteristics of patients with SUDs. However, this suggests that strategies are needed to improve engagement and retention in treatment with reSET-O.

One such strategy could be more integration between counseling sessions with clinical providers and the therapy modules delivered by the app. reSET-O is largely psychoeducational and didactic in nature. Future studies should invite counselors and medical providers to discuss reSET-O and the topics therein with patients to make the content more relevant and engaging. Clinicians prescribing medication routinely discuss medication adherence, side effects, and whether and how it may be helpful. Similarly, clinicians prescribing reSET-O could ask about what modules the patient has completed, troubleshoot adherence problems, and discuss how the patient is applying the therapy modules delivered by the app in their daily lives. In this way, in-person and mobile treatments can enhance one another to perhaps increase treatment retention. Some patients suggested additional praise or encouragement for reaching certain milestones, such as maintaining a certain number of favorable urines or reaching a specified length of time of sobriety, which could be further facilitated by counselors. A team approach among providers, patients, and reSET-O may better address any problems with adherence, perceived burden, and the relevance of the topics. The integration of remote therapy with in-person treatment might enhance the salience of reSET-O and reduce enthusiasm decay over time.

Second, provider feedback, although encouraging, provided some helpful suggestions on language. On the basis of the WIAAF scores, the feasibility of implementing reSET-O had the highest average score when compared with the other categories. The category with the lowest average score was appropriateness because of the content language. As noted above, some providers felt that the language appeared stigmatizing in some areas or was difficult to understand (eg, reading level). TES was created >15 years ago, and much has changed in the culture and lexicon surrounding SUDs. This presents a challenge in terms of how a digital therapeutic could be adjusted based on the patient population and setting, perhaps based on real-time feedback from local patients and providers, to provide a product that is maximally inviting and relevant. Regarding the scope of services, providers felt that emergency information might be beneficial to the patient. Mobile technology in this arena has been developed through the Addiction–Comprehensive Health Enhancement Support System [28], and perhaps, interapplication communication can broaden the scope to help more patients.

The monetary remuneration for participants was relatively low compared with traditional evidence-based CM programs [20,37]. reSET-O rewards negative USDs and module completions, with the idea that incentives will help patients participate in the therapy modules; thus, internalizing techniques of cognitive behavioral therapy can prevent ongoing drug use and address problems with cravings, mood, and relationships. Traditionally, CM has shown efficacy in rewarding individuals for appointment adherence and negative USDs alone. Moreover, larger rewards were available in prior grant-funded studies [38,39]. That reSET-O funds incentives through third-party reimbursement is an innovation that overcomes the problem of how to fund incentives and makes CM feasible in community-based practice. However, only modest reward values are possible using this approach. reSET-O, and its forerunner TES, use the *prize bowl* model for low-cost CM developed by Petry [37]. This turns CM into a game where rewards are determined by chance; some are just verbal (eg, *good job*), and most rewards have low monetary value, approximately US $5, with an occasional larger reward value, which keeps the overall costs of contingencies low across treatment episodes.

### Limitations

Limitations include that this study was an uncontrolled trial with a small sample size and a high attrition rate. This study is most useful for generating ideas for improving feasibility and acceptability; however, without a control group, it is not possible to evaluate effectiveness. Patients retained in the study were mostly abstinent over the course of the study, and mood ratings were in the mild range of severity, which is encouraging. However, it is not possible to evaluate treatment retention or patients’ individual trends in substance use and mood without a larger sample and control condition. The fact that many patients who were abstinent during the study were already abstinent at baseline suggests the importance of covarying for baseline in future trials. The sample comprised predominantly White men, reflective of the local patient population, affiliated with the one particular clinic in which the study was conducted. The providers did not systematically discuss reSET-O with the enrolled patients during clinical visits. Participants might achieve more therapeutic benefits from reSET-O if it is integrated more into in-person counseling.

### Conclusions

Prescription digital therapeutics, such as reSET-O, have the potential to bridge a gap in MOUD, which often prevents potential providers from prescribing these life-saving medications, namely by addressing the need to deliver medication in conjunction with behavioral counseling. For providers who practice without live behavioral counseling on site and within financially stressed clinical programs, a mobile app has the potential to expand a clinic’s ability to meet the needs of populations with SUDs. Larger controlled trials are warranted to evaluate whether this intervention improves the adherence to and outcome of MOUD in community-based treatment settings such as the Hub and Spoke model of care in which this pilot study took place. Future work should examine ways that clinicians can integrate the patients’ participation in an app such as reSET-O into the counseling of their patients in an effort to improve adherence to the use of the app and maximize its impact as a clinician extender.

## Appendix

Multimedia Appendix 1 Wiener Intervention Acceptability, Appropriateness, and Feasibility form.

## Abbreviations

CM: contingency management
CRA: Community Reinforcement Approach
IAFF: Intervention Acceptability Feedback Form
MOUD: medication for opioid use disorder
OTP: opioid treatment program
OUD: opioid use disorder
PTSD: posttraumatic stress disorder
SUD: substance use disorder
TES: Therapeutic Education System
TLFB: Timeline Follow-Back
WIAAF: Weiner Intervention Acceptability, Appropriateness, and Feasibility form
