# Supplementary material for: Patient- and Provider-Reported Experiences of a Mobile Novel Digital Therapeutic in People With Opioid Use Disorder (reSET-O): Feasibility and Acceptability Study
Source: JMIR Form Res. 2022 Mar 25;6(3):e33073. doi: 10.2196/33073 (PMC8994143; doi:10.2196/33073)
Supplement: Multimedia Appendix 1 [file formative_v6i3e33073_app1.docx]

GENERAL INSTRUCTIONS: These measures could be used independently or together. The IAM items could be modified to specify a referent organization, situation, or population (e.g., my clients). Please check and report the psychometric properties with each use or modification.

**Acceptability of Intervention Measure (AIM)**

|  | Completely disagree | Disagree | Neither agree nor disagree | Agree | Completely agree |
| --- | --- | --- | --- | --- | --- |
| 1. (INSERT INTERVENTION) meets my approval. | ➀ | ➁ | ➂ | ➃ | ➄ |
| 2. (INSERT INTERVENTION) is appealing to me. | ➀ | ➁ | ➂ | ➃ | ➄ |
| 3. I like (INSERT INTERVENTION). | ➀ | ➁ | ➂ | ➃ | ➄ |
| 4. I welcome (INSERT INTERVENTION). | ➀ | ➁ | ➂ | ➃ | ➄ |

**Intervention Appropriateness Measure (IAM)**

|  | Completely disagree | Disagree | Neither agree nor disagree | Agree | Completely agree |
| --- | --- | --- | --- | --- | --- |
| 1. (INSERT INTERVENTION) seems fitting. | ➀ | ➁ | ➂ | ➃ | ➄ |
| 2. (INSERT INTERVENTION) seems suitable. | ➀ | ➁ | ➂ | ➃ | ➄ |
| 3. (INSERT INTERVENTION) seems applicable. | ➀ | ➁ | ➂ | ➃ | ➄ |
| 4. (INSERT INTERVENTION) seems like a good match. | ➀ | ➁ | ➂ | ➃ | ➄ |

**Feasibility of Intervention Measure (FIM)**

|  | Completely disagree | Disagree | Neither agree nor disagree | Agree | Completely agree |
| --- | --- | --- | --- | --- | --- |
| 1. (INSERT INTERVENTION) seems implementable. | ➀ | ➁ | ➂ | ➃ | ➄ |
| 2. (INSERT INTERVENTION) seems possible. | ➀ | ➁ | ➂ | ➃ | ➄ |
| 3. (INSERT INTERVENTION) seems doable. | ➀ | ➁ | ➂ | ➃ | ➄ |
| 4. (INSERT INTERVENTION) seems easy to use. | ➀ | ➁ | ➂ | ➃ | ➄ |

Pragmatic Qualities:

- Readability tested by substituting “This EBP” for “Insert Intervention.” Flesch reading ease score (and grade level) is 95.15 (5^th^ grade) for AIM, 99.60 (5^th^ grade) for IAM, and 94.17 (5^th^ grade) for FIM.
- No specialized training is needed to administer, score, or interpret the measures.
- Cut-off scores for interpretation not yet available; however, higher scores indicate greater acceptability, appropriateness, or feasibility.
- Norms not yet available.
- Scales can be created for each measure by averaging responses. Scale values range from 1 to 5. No items need to be reverse coded. Good measurement practice: assess structural validity to confirm the unidimensionality of each measure and calculate alpha coefficient to ascertain reliability.
- There is no cost to use these measures.
- Time to complete: less than 5 minutes per measure.
